# Fermented feed-rumen microbiota axis mediates goat kid growth via microbial amino acid synthesis and ruminal epithelial energy metabolism

**DOI:** 10.1038/s41522-026-01046-z

**Published:** 2026-06-11

**Authors:** Fanlin Kong, Fenghui Liu, Yong Qian, Ziyu Wang, Wenfeng Zhang, Yan Wang, Jianli Zhang, Yinxia Li, Jun Zhang, Shuo Wang, Dongwen Dai, Zhiping Lin, Huili Wang, Chunhua Meng

**Affiliations:** 1 Institute of Animal Science, Jiangsu Academy of Agricultural Sciences, Nanjing, China; 2 Key Laboratory of Crop and Animal Integrated Farming, Ministry of Agriculture and Rural Affairs, Nanjing, China; 3 Jiangsu Provincial Engineering Research Center of Precision Animal Breeding, Nanjing, China; 4 College of Animal Science, Nanjing Agricultural University, Nanjing, China; 5 Inner Mongolia Agricultural University, Key Laboratory of Dairy Biotechnology and Engineering, Ministry of Education, Hohhot, China; 6 Academy of Animal Science and Veterinary, Qinghai University, Xining, China; 7 Nanjing Youyuan Dairy Research Institute Co. Ltd., Nanjing, China

**Keywords:** Biotechnology, Microbiology

## Abstract

Pretreating feed via fermentation to enhance its nutritional value is effective for young ruminants. This study optimized fermented feed using a microbial consortium of *Saccharomyces cerevisiae*, *Bacillus licheniformis*, and *Enterococcus faecalis*, and explored its mechanisms on growth performance and ruminal function in goat kids. The *Bacillus licheniformis* is the most critical determinant of the nutritional value of FF. Optimized FF had higher levels of crude protein, calcium, phosphorus, and limiting amino acids (AA). The FF contained viable microbiota with lower diversity but greater evenness. The microbiota was enriched in AA and carbohydrate metabolism by enhancing the abundance of *Leuconostoc lactis* and *Lactococcus lactis*. A 40-day feeding trial was then conducted with 20-day-old goat kids. Compared to conventional feed, FF increased feed digestibility, body weight and carcass weight. Mechanistically, FF enhanced the interactions among rumen microbes and increased the gene abundance related to carbohydrate and AA metabolism. The FF then upregulated expression of mitochondrial respiratory chain-related genes in the rumen epithelium, thereby improving epithelial energy metabolism. Overall, this study shows that fermented feed optimized by microbial consortium improves goat kid growth and carcass quality through the fermented feed–rumen microbiota axis, offering a sustainable approach for ruminant production.

**Figure:**
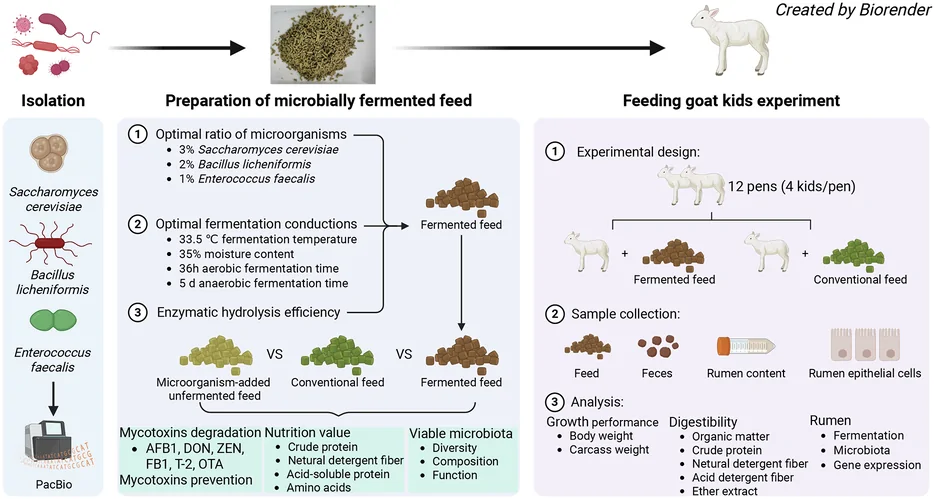

## Introduction

Food insecurity refers to the lack of access to food, as well as the nutritional quality and safety of the food. In 2024, about 2.3 billion people experienced moderate or severe food insecurity, which is 336 million more than in 2019^1^. Intensive livestock production consumes about one-third of the world’s grains and two-thirds of soybeans, corn, and barley^2^. There is competition for essential resources, such as feed, water, and land, between livestock production and human food production, with ruminants being a particular focal point of this contention^3^. Leveraging grazing and incorporating by-products into ruminant feed can alleviate the burden on food resources associated with livestock production^4^. Additionally, processing diets to enhance feed utilization efficiency offers a fundamental way to reduce this burden, such as through microbial pretreatment^5^. It involves the hydrolysis of complex organics into simpler small molecules.

*Saccharomyces cerevisiae*, *Bacillus licheniformis*, and *Enterococcus faecalis* are common strains used in ruminant feed, and they are added for two primary purposes: firstly, for feed pretreatment^6–8^, and secondly, for optimizing the gastrointestinal environment of animal^9–13^. Among these, *Bacillus licheniformis* possesses a unique biological oxygen-scavenging mechanism that facilitates the rapid establishment of an anaerobic environment^14^, while also producing antibacterial active substances to inhibit the growth and proliferation of pathogenic bacteria^6^. Also, *Bacillus licheniformis* exhibits robust activities of protease, lipase, and amylase, which promote the degradation of nutrients in feed, especially complex carbohydrates, thereby enhancing the efficiency of feed absorption and utilization by animals^8^. *Saccharomyces cerevisiae* leverages its efficient sugar metabolic capacity and its ability to synthesize metabolites (e.g., enzymes and AA)^15^. It acts synergistically with strains of the *Bacillus* genus, exhibiting remarkable efficacy in cellulose degradation and feed palatability enhancement^7^. *Enterococcus faecalis*, a common probiotic in feed, aids diet fermentation by producing lactic acid via homofermentation^16^. It lowers pH to inhibit pathogens and activate hydrolases for substrate degradation, and also secretes peptidases to break proteins into free amino acids, enhancing feed nutrition^16^. Currently, most studies focus on single-strain fermentation for feed pretreatment, while research exploring the synergistic effects of combined fermentation remains scarce. Combined fermentation has also exhibited excellent effects in aspects such as liquor-making^17^ and conversion of lignocellulosic feedstock^18^.

Beyond its application in fermented feed, studies currently also opt for the direct feeding of probiotics to ruminants^11,15,19^. The rumen microbiota structure of adult ruminants is stable, and exogenous probiotics often fail to colonize in the rumen^20^. Hence, feeding probiotics yields favorable outcomes in young ruminants, primarily due to the fact that their rumen microbiota is in the establishment stage^9,11,19^. On one hand, probiotics can secrete digestive enzymes in the rumen to promote the digestion and absorption of complex carbohydrates or proteins^21,22^. It is of great significance for young ruminants with immature digestive system development^23^. On the other hand, they can inhibit the colonization of pathogenic bacteria by secreting antimicrobial peptides and bacteriocins, creating an acidic environment, and competing for ecological niches^24,25^. This is of great importance for young ruminants that are in the process of intestinal microbiota establishment and have immature immune functions^23^. However, the effect of microbial co-fermentation on the composition of viable microorganisms in fermented feed remains unknown. Furthermore, it is unclear whether the organisms in fermented feed can effectively colonize the rumen of young ruminants or benefit rumen fermentation.

We hypothesize that the co-fermentation of *Saccharomyces cerevisiae*, *Enterococcus faecalis*, and *Bacillus licheniformis* has a synergistic effect in enhancing dietary nutrients and increasing beneficial microbiota in the diet. These effects together regulate rumen fermentation and promote growth in young ruminants. To test this hypothesis, we first isolated and screened rumen-derived *Saccharomyces cerevisiae*, *Enterococcus faecalis*, and *Bacillus licheniformis* from the rumen fluid of adult ruminants. Subsequently, orthogonal experiments were designed to identify the optimal strain ratio and fermentation conditions for dietary fermentation. Then, metatranscriptomic sequencing was used to investigate whether these three strains could successfully colonize the fermented feed and their subsequent impacts on the feed’s microbial composition. Finally, goat kids were fed either fermented or unfermented diets to assess the effects of the fermented diet on rumen fermentation, rumen epithelial function, slaughter performance, and overall growth performance. This study aims to develop a microbial fermentation strategy that leverages the additive effects of these three strains for diet co-fermentation, providing a scientific basis for improving resource use efficiency.

## Results

### Determination of optimal parameters of fermented feed

**Fig. 1:**
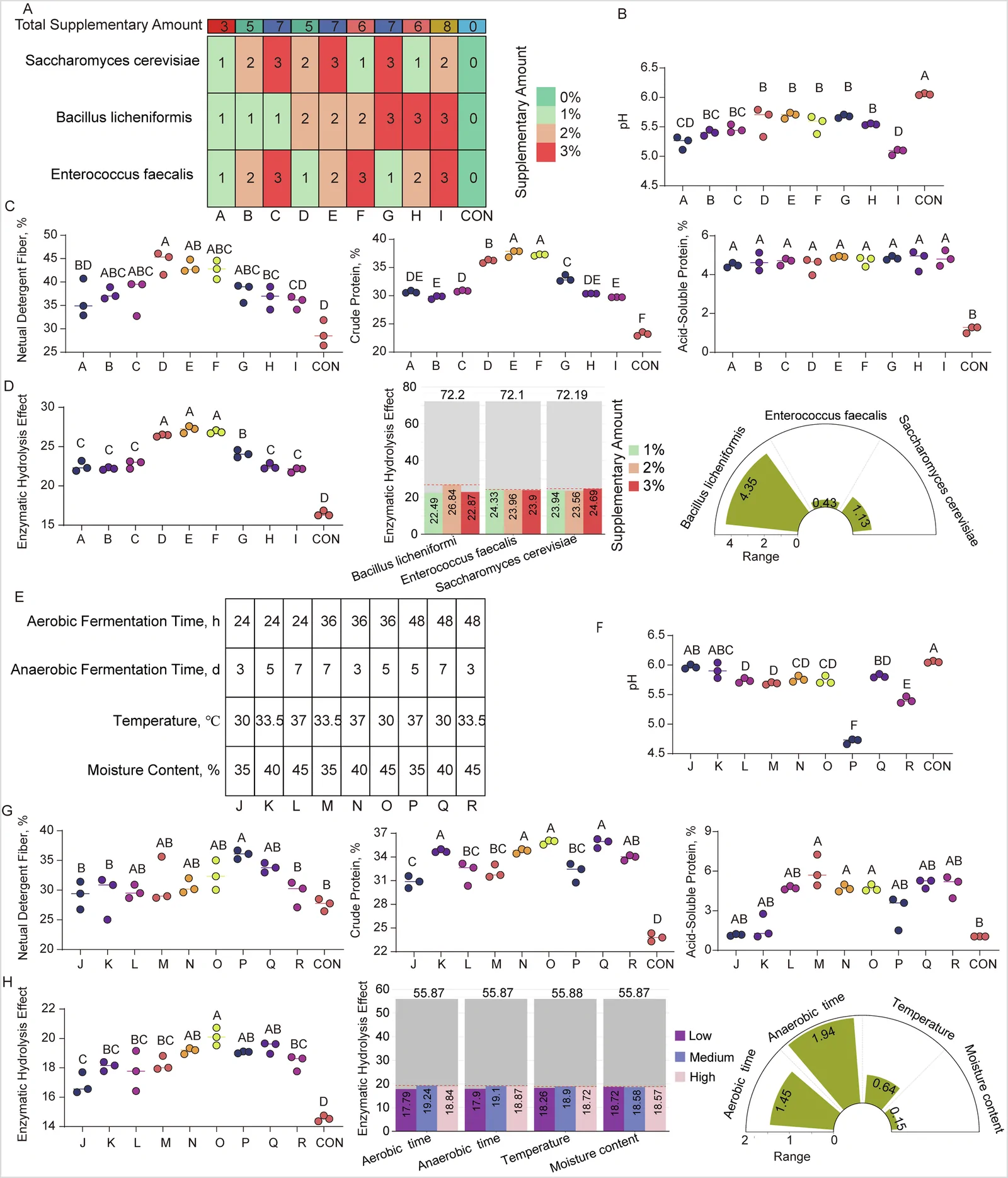
Optimization of Fermentation Conditions for Fermented Feed. Determination of optimal fermentation ratios (**A**–**D**) and conditions (**E**–**H**) for *Saccharomyces cerevisiae*, *Enterococcus faecalis*, and *Bacillus licheniformis* co-fermentation in fermented feed. Statistical significance was calculated using one-way ANOVA with Tukey multiple comparisons. Different letters indicate significant differences (*P* < 0.05).

Figure 1A shows the experiment design for determining the optimal ratio of microorganisms. The pH values of all fermented feed were significantly lower than CON group (Fig. 1B) and the CP and AP content were significantly higher than CON group (Fig. 1C). After calculating the E value, the E values of all group were considerably higher than CON group (Fig. 1D). Among the three addition levels for each strain, the mean E value was calculated for all groups sharing the same level (Fig. 1D). The 2% addition level of *Bacillus licheniformis*, the 1% addition level of *Enterococcus faecalis*, and the 3% addition level of *Saccharomyces cerevisiae* yielded the highest mean E values within their respective concentration gradients (Fig. 1D, Supplementary Table 1). The E value range of *Bacillus licheniformis* was higher than that of the other microorganisms (Fig. 1D, Supplementary Table 1).

Figure 1E shows the experiment design for determining optimal fermentation conditions. The pH values of the L-R groups were significantly lower than those of the CON group (Fig. 1F). The NDF content of the P group was significantly higher than that of the CON group, and the CP content of all groups was significantly higher than that of the CON group (Fig. 1G). Then, the mean E value for each level of different fermentation parameters was calculated and compared (Fig. 1H). Among these conditions, 36 h of aerobic fermentation time, 5 d of anaerobic fermentation time, 33.5°C of fermentation temperature, and 35% moisture content exhibited the highest mean E values within their respective parameter gradients (Fig. 1H, Supplementary Table 2). Finally, the E value range of anaerobic fermentation time was higher than the other conditions (Fig. 1H, Supplementary Table 2).

### Nutritional advantages of fermented feed

**Fig. 2:**
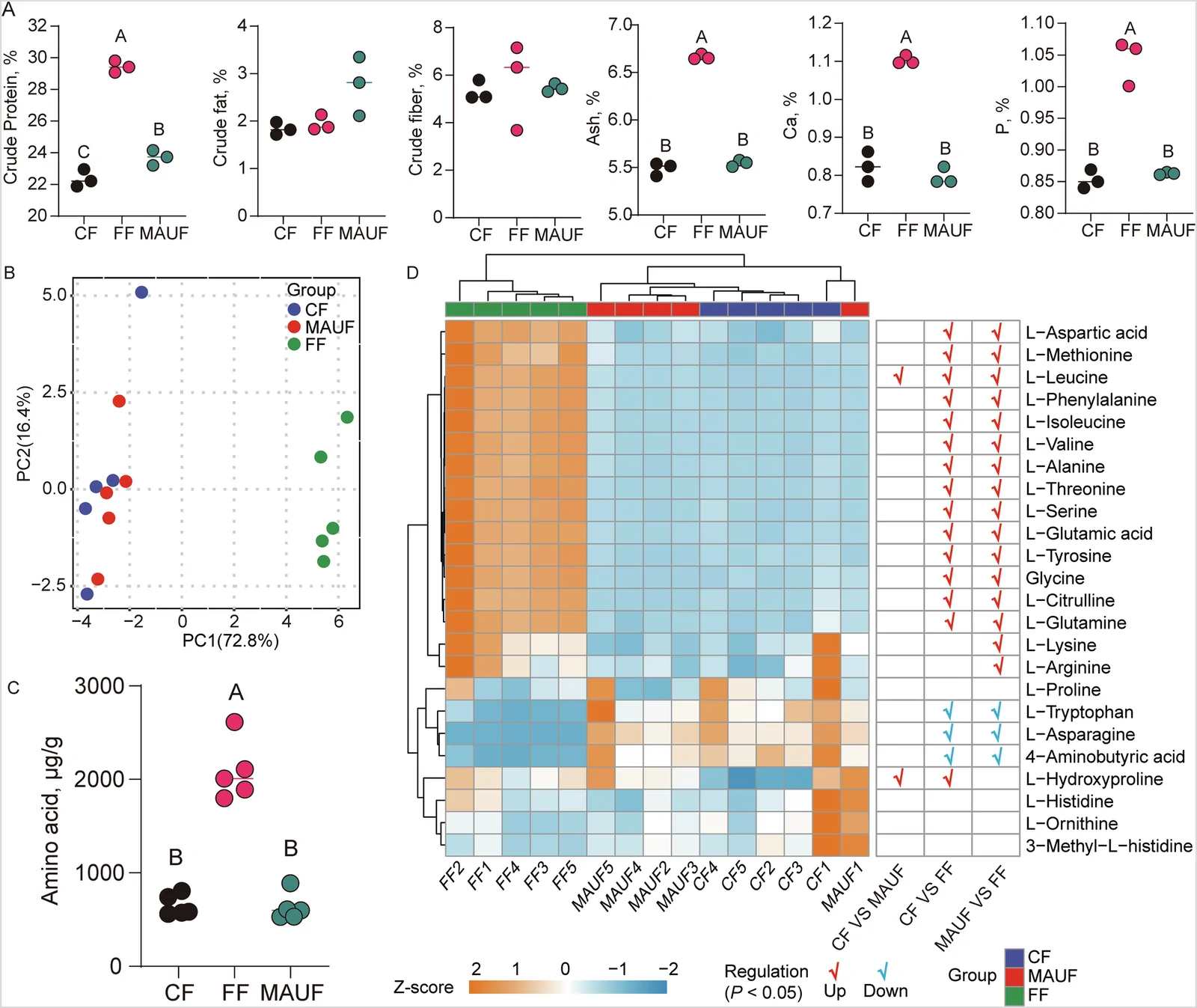
Superiority in Nutritional Composition of Fermented Feed. Comparison of conventional nutritional components (**A**) and amino acid concentrations (**B**–**D**) among fermented feed (FF), conventional feed (CF), and microorganism-added unfermented feed (MAUF). Statistical significance was calculated using one-way ANOVA with Tukey multiple comparisons. Different letters indicate significant differences (*P* < 0.05).

After the fermentation conditions were established, we compared the nutritional compositions of FF with CF and MAUF to emphasize the advantages of FF (Fig. 2). The CP, Ash, Ca, and P contents of FF were significantly higher than those of CF and MAUF (Fig. 2A). The CP content of MAUF was higher than that of CF (Fig. 2A). Figure 2B shows that the AA composition of FF is significantly distinguished from other groups. The total AA concentrations of FF were significantly higher than those of CF and MAUF groups (Fig. 2C). Finally, the concentrations of 14 AA in the FF group were significantly higher than those of CF and MAUF (Fig. 2D). The concentrations of Tryptophan, Asparagine, and 4-Aminobutyric acid in the FF were significantly lower than those of CF and MAUF (Fig. 2D).

### Changes in viable microbiota composition in feed after microbial fermentation

**Fig. 3:**
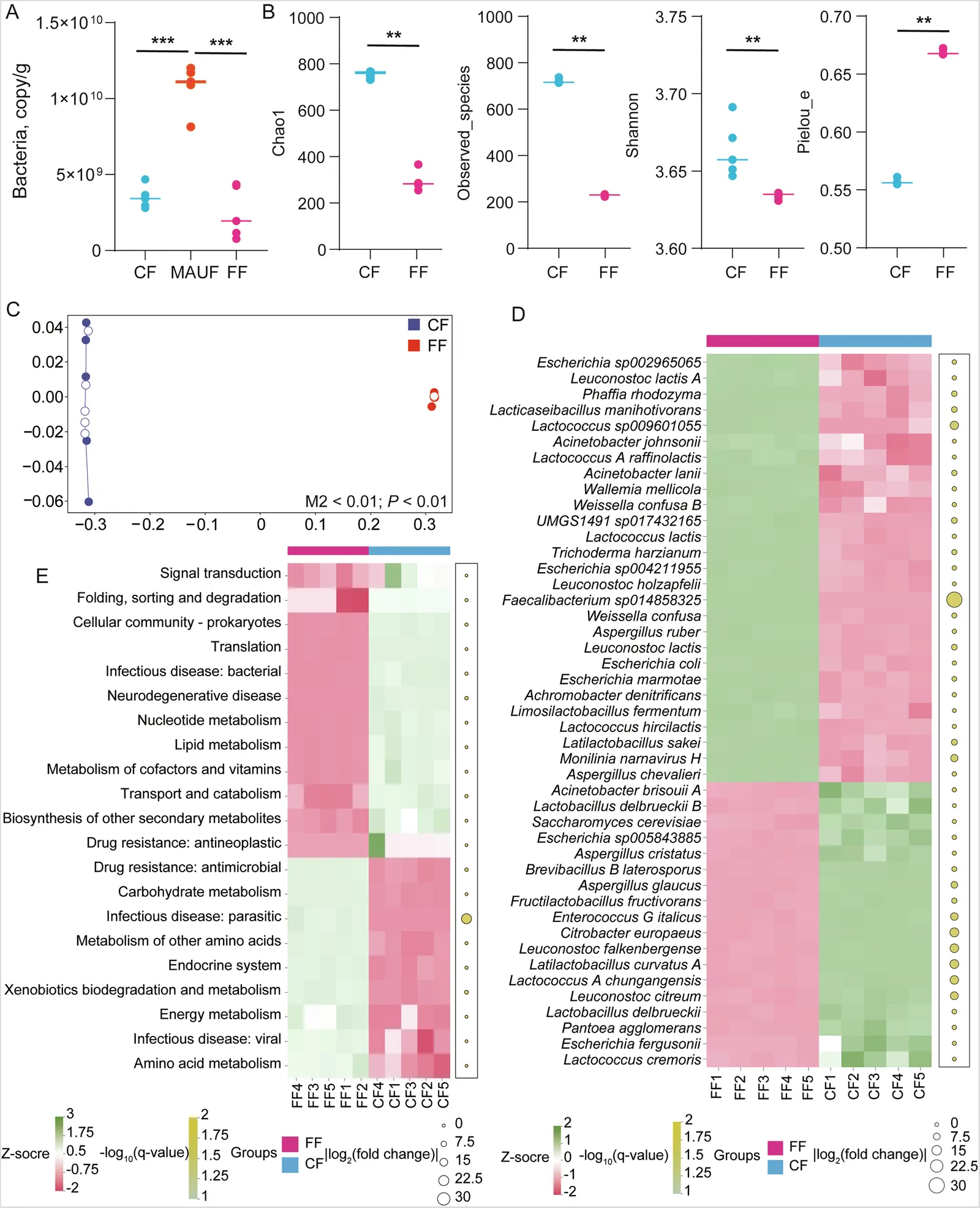
Comparison of viable microbial composition and diversity between fermented feeds (FF) and conventional feeds (CF). **A** copy number of bacteria; **B** α diversity indices of viable microbiota in the CF and FF; **C** Procrustes analysis of microbiota composition and functions between CF and FF; **D** significantly different KEGG pathways on level 2; **E** significantly different species. *P < 0.05; **P < 0.01; ***P < 0.001.

In addition to the nutritional value, we also detected the viable microbiota composition of FF. Firstly, the AQ of bacteria shows that the copy number in the MAUF was significantly higher than in the CF and FF groups. The copy number of FF was lower than that in the CF (Fig. 3A). Also, the Chao1, species number, and Shannon of FF were significantly lower than those in the CF. At the same time, the Pielou evenness index in the FF was significantly higher than in CF (Fig. 3B). Procrustes analysis shows that the distribution of microbiota composition and function was significantly separated between CF and FF, and the M2 value was lower than 0.01 (Fig. 3C). The NCM shows that the R2 of CF was 0.594. The NCM of FF did not meet the criteria because the model fit R² was less than 0.1 (Supplementary Fig. 1).

Then, the abundances of microbiota involving in carbohydrate metabolism, AA metabolism, energy metabolism were higher in the FF when compared with CF, while the abundances of microbiota involving in infectious disease were also higher than CF (Fig. 3D). Specifically, the abundance of *Saccharomyces cerevisiae* in the FF was significantly lower than that in the CF (Fig. 3E) and the detailed information of significant different species (SDS) was presented in Supplementary Table 3.

**Fig. 4:**
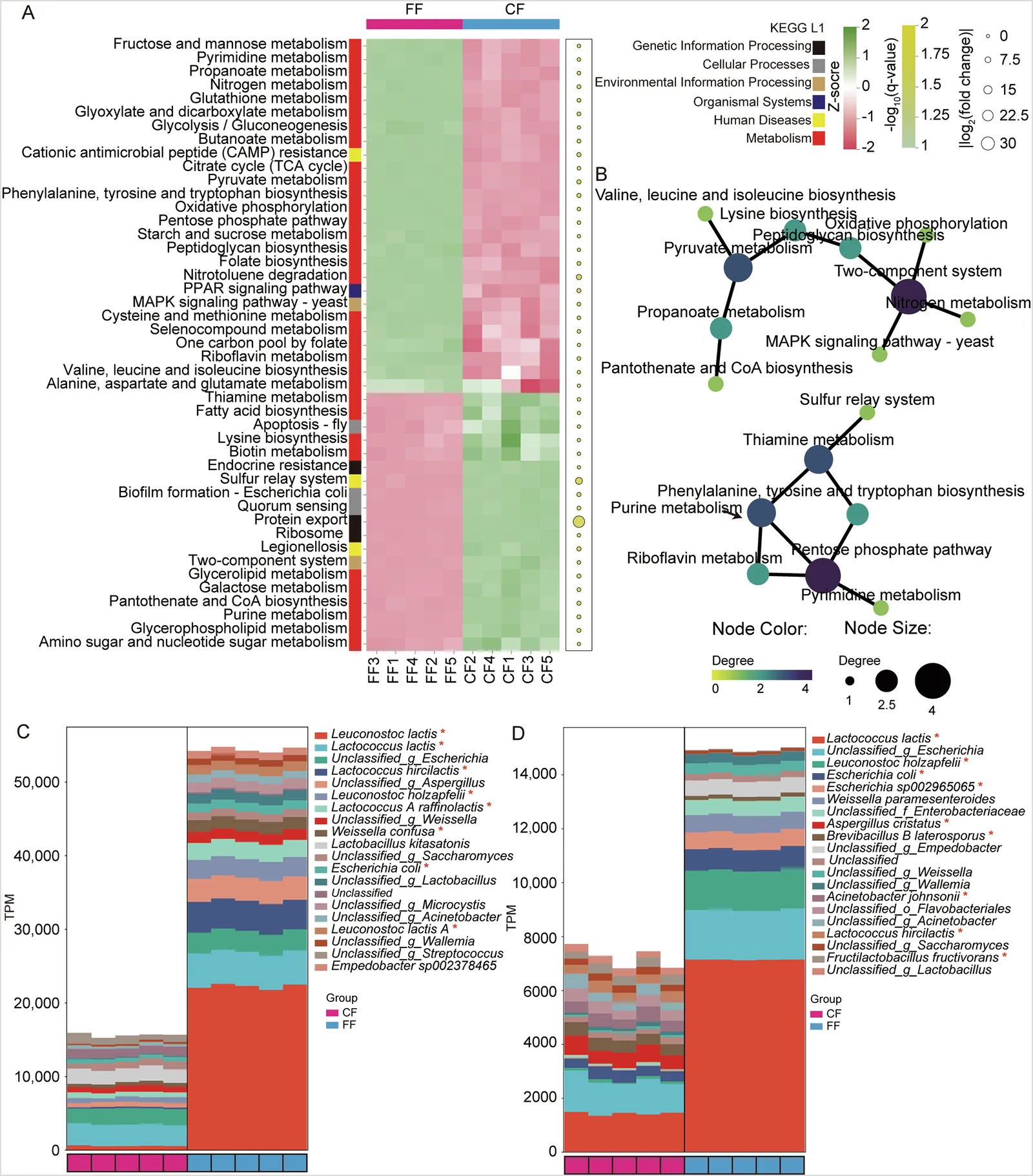
Comparison of viable microbial function between fermented feeds (FF) and conventional feeds (CF). **A** Functional differences between FF and CF; the relationship of significant changed pathways from metatranscriptomics; **B** Upstream and downstream relationships of differential KEGG pathways; the key microorganisms involved in carbohydrate (**C**) and amino acid metabolism (**D**). Red asterisks indicate microbes with significant differences between the two groups (*P* < 0.05). *P* values were calculated using the two-tailed Wilcoxon rank-sum test.

Then, we compared the significantly different KEGG level 3 pathways (Fig. 4A). Most pathways were derived from Metabolism. Several AA, including Phe, Tyr, Trp, Gln, Cys, Met, Val, Leu, Ile, Ala, Asp, and Glu metabolism and nitrogen metabolism were enriched in FF. Carbohydrate metabolism, including fructose, mannose, pyruvate, pentose phosphate, starch, sucrose, and folate metabolism, was enriched in FF. Figure 4B shows that the significantly different pathways have a close relationship between upstream and downstream metabolism. Fig. 4C and D show the microbiota genes involved in carbohydrate metabolism (Fig. 4C) and AA metabolism (Fig. 4D). The species, including *Leuconostoc lactis*, *Lactococcus lactis*, and *Lactococcus hircilactis*, were enriched in carbohydrate metabolism and significantly higher in the FF (Fig. 4C). The species, including *Lactococcus lactis* and *Leuconostoc holzapfelii*, were enriched in AA metabolism and significantly higher in the FF (Fig. 4D).

### Effects of FF feeding on growth performance and rumen microbiota in kids

**Fig. 5:**
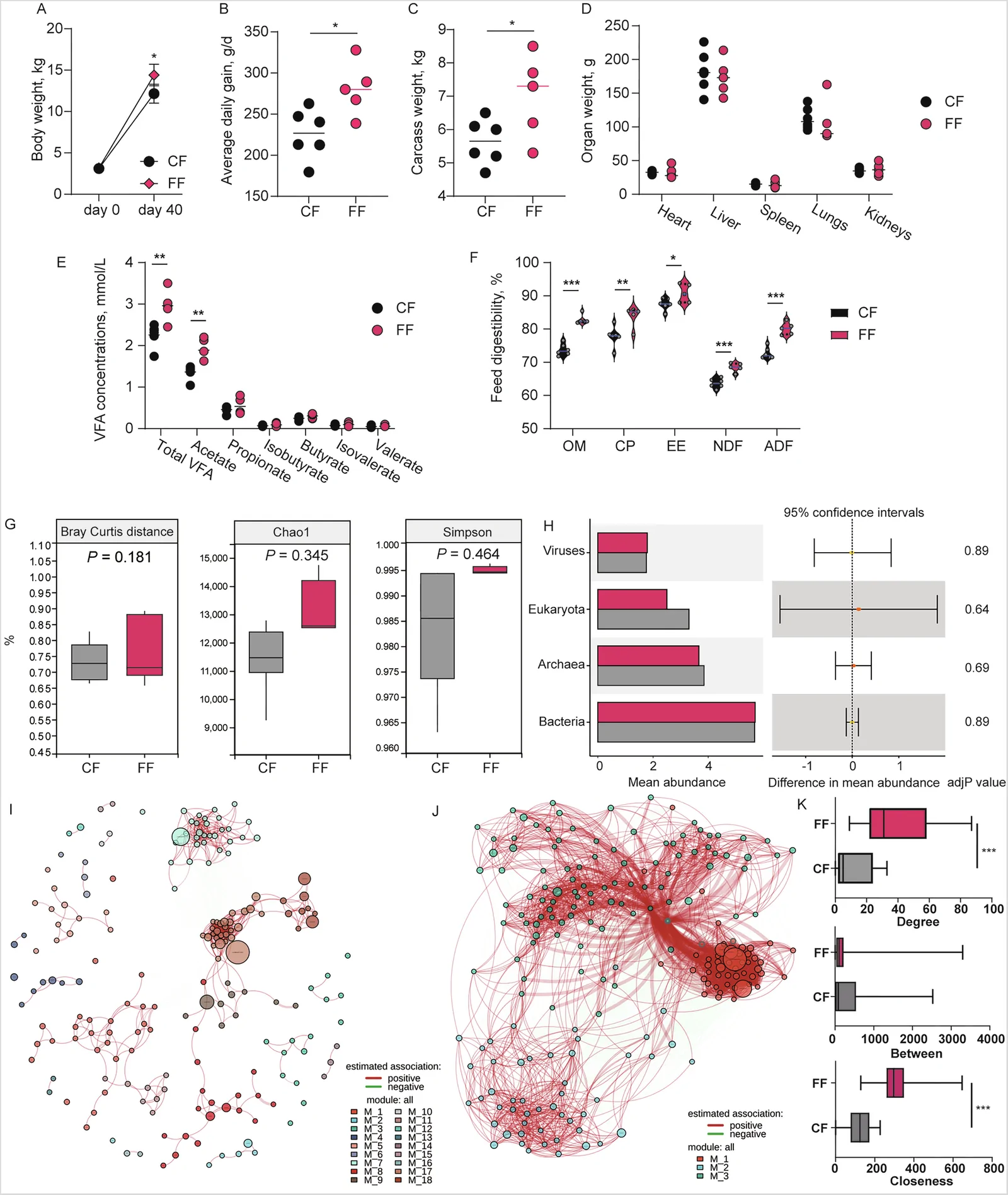
Effects of FF feeding on growth performance and rumen microbiota in goat kids. **A**, **B** growth indices; **C**, **D** carcass indices; **E** volatile fatty acid concentration; **F** feed digestibility; **G** α diversity indices of rumen microbiota; **H** microbiota composition at the domain level; **I** microbial interaction network of rumen microbiota on the CF group; **J** microbial interaction network of rumen microbiota on the FF group; **K** network indices of rumen microbiota on the CF and FF groups. *P < 0.05; **P < 0.01; ***P < 0.001.

After 40 days of feeding, the BW and ADG of the FF groups were significantly higher than those of kids in the CF group (Fig. 5A, B). Additionally, the carcass weight of kids in the FF group was significantly higher than that of the CF group (Fig. 5C). The organ weight, length of the small intestinal segments, weight of the ruminant stomach, and volume of the ruminant stomach did not significantly differ between the CF and FF groups (Fig. 5D, Supplementary Fig. 2). Furthermore, the total VFA and acetate concentrations in the FF group were significantly higher than those in the CF group (Fig. 5E). The OM, CP, EE, NDF and ADF digestibility were significantly higher in the FF group than those in the CF group (Fig. 5F).

Then, we measured the rumen microbiota, and the diversity of rumen microbiota was not different between the two groups (Fig. 5G). Additionally, the composition of rumen microbiota at the domain level also remained unchanged between the two groups (Fig. 5H). The results of NCM show that the R^2^ value of the CF group was higher than that of the FF group (Supplementary Fig. 3). Microbial interaction network analysis in Fig. 5I and J indicates that interspecific interactions were more abundant and more connected. After calculating the network indices, the degree and closeness of the network in the FF group were significantly higher than those in the CF group (Fig. 5K).

**Fig. 6:**
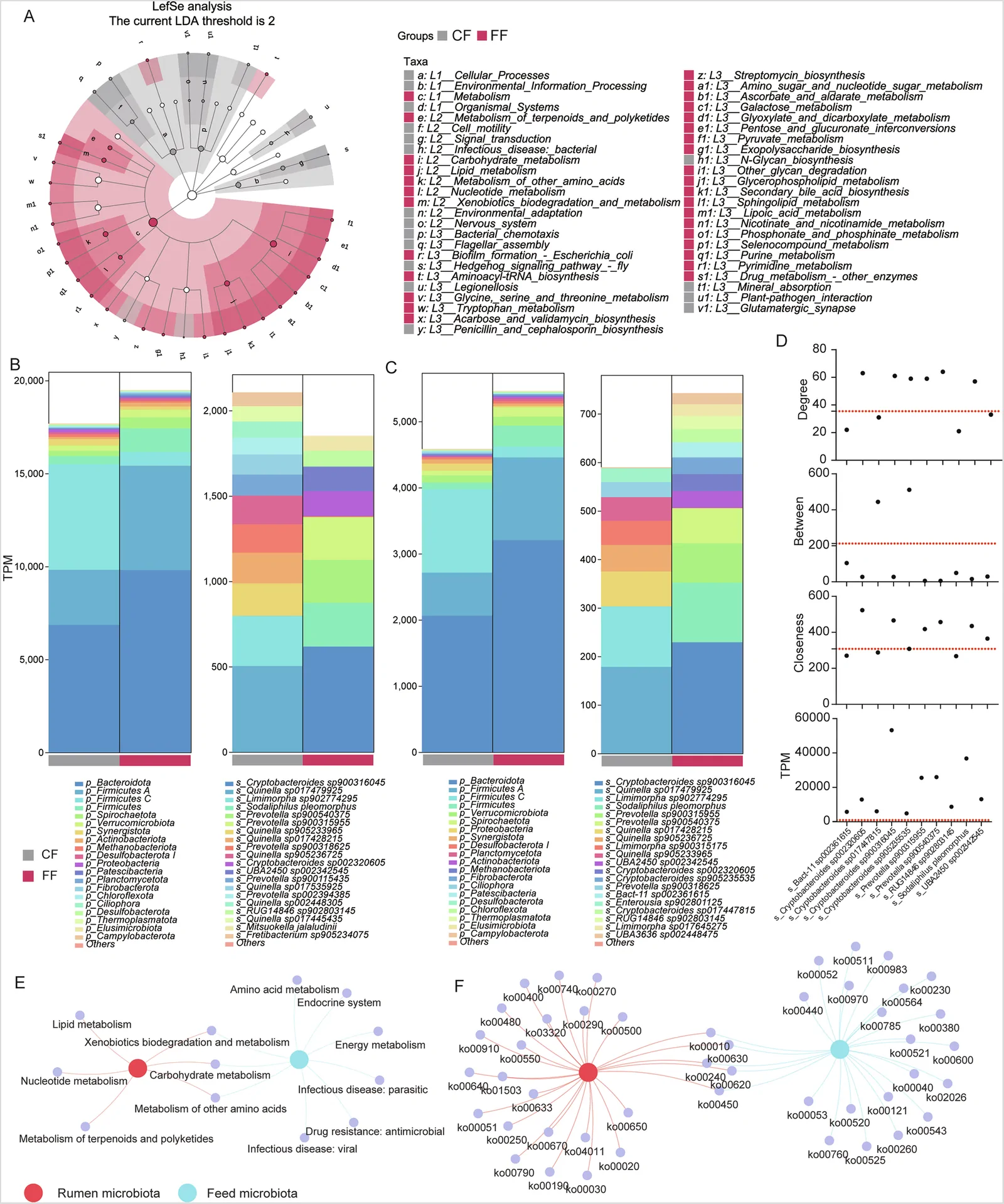
Comparison of rumen microbial functional differences in goat kids fed FF and CF. **A** LefSe analysis to compare the significantly different microbial functions between FF and CF kids; **B** phylum and species in the CF and FF groups which contributed to carbohydrate metabolism; **C** phylum and species in the CF and FF groups which contributed to lipid metabolism; **D** comparison of microbial network indices between species contributing to carbohydrate and lipid metabolism and other species. The red line indicates the average of the network indices of other species. **E** the common significantly different KEGG L2 pathways between the feed microbiota and the rumen microbiota. **F** the common significantly different KEGG L3 pathways between feed microbiota and rumen microbiota.

Then, we analyzed the SDS and functions between the CF and FF groups (Fig. 6), and the Metabolism pathway was enriched in the FF group. KEGG level 2 pathways, including carbohydrate metabolism, lipid metabolism, metabolism of other AA, and nucleotide metabolism, were enriched in the FF group (Fig. 6A). Considering the feed degradation of rumen microbiota, Fig. 6B and C show the dominant phylum contributing to carbohydrate and lipid metabolism, including Bacteroidota and Firmicutes A in both groups. Specifically, Firmicutes C in the CF group contributed mainly to carbohydrate and lipid metabolism and was different from the FF group (Fig. 6B, C). Furthermore, the compositions of species that contributed to carbohydrate and lipid metabolism were different between the two groups. The dominant species were *Quinella sp017479925*, *Limimorpha sp902774295*, and *Quinella sp017428215* in the CF group (Fig. 6B). The dominant species were *Cryptobacteroides sp900316045*, *Sodaliphilus pleomorphus*, and *Prevotella sp900315955* in the FF group (Fig. 6C). Finally, we examined the role of species in the microbiota network of the FF group, which contributed to both carbohydrate and lipid metabolism and TPM > 1. Compared to the mean of other species in the FF network, most species contributing to carbohydrate and lipid metabolism in the FF group had a higher degree and closeness, and their TPM were higher than those of other species (Fig. 6D).

The common pathways between significantly different pathways of feed microbiota (Figs. 3D, 4A) and significantly different pathways of rumen microbiota (Fig. 6A). The common pathways on the KEGG L2 were Carbohydrate metabolism, Xenobiotics biodegradation and metabolism, and Metabolism of other AA (Fig. 6E). The common pathways on the KEGG L3 were Glycolysis/Gluconeogenesis, Glyoxylate and dicarboxylate metabolism, Pyrimidine metabolism, Pyruvate metabolism, and Selenocompound metabolism (Fig. 6F).

**Fig. 7:**
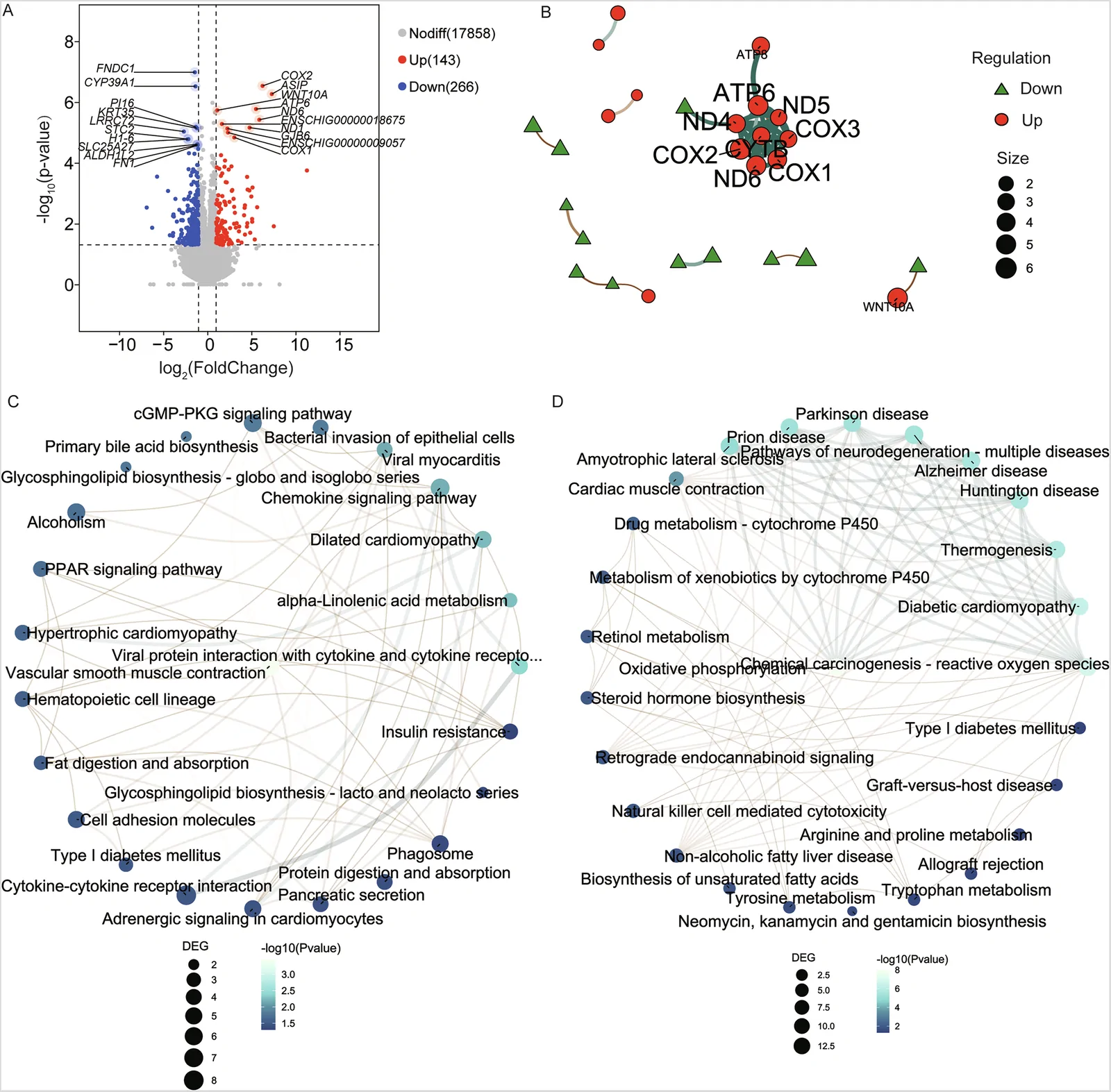
Effects of FF feeding on gene expression of rumen epithelial cells in goat kids. **A** the summary of significantly different genes; **B** the protein-protein interaction network analysis of significantly different genes; **C** the down-regulated pathways in the FF group when compared with the CF group; **D** the up-regulated pathways in the FF group when compared with the CF group.

Finally, we measured gene expression in rumen epithelial cells (Fig. 7). Figure 7A displays the genes significantly different between the CF and FF groups, including 143 upregulated genes and 266 downregulated genes. Next, the protein-protein interaction network analysis shows that ATP6, ND4, ND5, ND6, COX3, COX2, COX1, and CYTB form a sub-network (Fig. 7B). When assigning the up- or down-regulated genes to KEGG pathways, pathways such as PPAR signaling, fat digestion and absorption, vascular smooth muscle contraction, and cytokine-cytokine receptor interaction were down-regulated (Fig. 7C). The up-regulated pathways included oxidative phosphorylation, biosynthesis of unsaturated fatty acids, arginine and proline metabolism, tyrosine metabolism, and tryptophan metabolism (Fig. 7D).

## Discussion

The present study aimed to ferment ruminant feed using a combined fermentation biotechnology of *Bacillus licheniformis*, *Enterococcus faecalis*, and *Saccharomyces cerevisiae*, with the goals of improving the nutritional value of the diet, ultimately applying FF in kids to confer beneficial effects on their growth performance, and finally enhancing resource utilization efficiency. Firstly, we found the clearly positive effects of fermented feed, whether single- or multiple-microorganism, compared with CF. It emphasized the potential role of fermented feed on enhancing the resource utilization efficiency again^17,26^. Regarding the safety of *Enterococcus faecalis* in this study, its pathogenicity is highly strain-dependent. Our strain, isolated from the rumen of healthy Qingshan goats, likely lacks key virulence genes and exhibits no hemolytic activity or drug resistance. It also aligns with observations in healthy human-derived *Enterococcus faecalis* strains^27^. The *Bacillus licheniformis* was the most impactful microorganism on feed nutrition. Previous studies have demonstrated the persistent stress resistance and stability of *Bacillus licheniformis* in fermentation systems when compared with other candidate strains for feed fermentation^25,28^. It not only possesses a unique biological oxygen-scavenging mechanism to establish an anaerobic environment rapidly but also promotes the preliminary decomposition of feed nutrients^8^, thereby providing sufficient growth substrates and a suitable ecological environment for the proliferation and synergistic fermentation of *Enterococcus faecalis* and *Saccharomyces cerevisiae*^11,29^. Hence, special attention should be paid to *Bacillus licheniformis* during the establishment of fermented feed systems.

After fermentation, the concentrations of CP and free AA were enhanced in the FF. This result is consistent with both the preliminary expectations and previous research findings. Qi et al.^8^ found that Bacillus licheniformis could increase the degree of protein hydrolysis of soybean meal, but the decomposition efficiency was limited. The preliminary decomposition of feed by *Bacillus licheniformis* provides abundant nutrient substrates and a favorable growth environment for the proliferation of *Enterococcus faecalis* and *Saccharomyces cerevisiae*. Conversely, the anaerobic environment established by *Bacillus licheniformis* optimizes *Enterococcus faecalis* lactic acid production, and the lactic acid further enhances the hydrolytic enzyme activity of *Bacillus licheniformis*^14^. Additionally, the bacteriocins produced by *Enterococcus faecalis* synergize with the antibacterial substances of *Bacillus licheniformis* to expand the pathogen inhibition spectrum^27,30^, improving the stability of the fermentation system. This may also be the reason for the decrease in microbial diversity index in the fermented feed of this study. Additionally, the acidic environment created by *Bacillus licheniformis* and *Enterococcus faecalis* regulates the metabolism of *Saccharomyces cerevisiae* toward the synthesis of beneficial metabolites, including enzymes and AA precursors, rather than ethanol production^31^. This is consistent with the results of previous studies on the synergistic fermentation of *Bacillus licheniformis* and *Saccharomyces cerevisiae*^11^. The microbial combination used in this experiment also coexists in various fermentation environments, thereby confirming a syntrophic relationship, such as in soybean meal-fermented feed^29^ and ben saalga^32^.

Although combined fermentation effectively improved protein and AA concentrations, particularly increasing the concentrations of various essential AA for animals, it did not reduce crude fiber content. This may be attributed to the simultaneous decrease in the total dry matter and fiber contents of the fermentation substrate, as the consumption of partially soluble carbohydrates during fermentation lowered the substrate’s total dry matter content. It was further corroborated by the increased ash content (as fermentation does not alter total ash amount)^16^. The CP content was increased by approximately 6%, which was higher than the results reported in a previous single-strain fermentation study and comparable to the efficacy of enzyme‑microbe combined fermentation^8,24^. In corn-soybean meal-based fermented feed, the improvement in crude protein content obtained via dual-strain synergistic fermentation was also inferior to that in the present study, with an increase of only approximately 4%^16^. Hence, our experiment revealed that the combined fermentation of *Bacillus licheniformis*, *Enterococcus faecalis*, and *Saccharomyces cerevisiae* synergistically enhanced the protein content and nutritional value of the feed.

Researchers have conducted work on FF and direct live microorganism feeding for the last few decades; however, few studies have evaluated the residual composition of beneficial organisms in the feed after fermentation^29,33^. Although individual kid DMI was unavailable, pen-level DMI showed minor differences between groups. DMI in the fermented feed group was lower, likely due to its higher moisture content, indicating kid weight gain in this group was not attributed to DMI. Our study found that microbiota diversity decreased after fermentation, consistent with a previous study^33^. This may be associated with the presence of artificially added fermentative microorganisms in the feed, which occupy a dominant position. Then, the evenness of microbiota increased after fermentation, and the results were consistent with the previous fermentation studies^34^. The experimentally added strains were not detected among the dominant bacteria, indicating that the added strains are transient in the feed fermentation process. They may promote fermentation, but eventually disappear. This also explains why the abundance of fermentation-added strains did not increase in the rumen of kids after they were fed fermented feed. Furthermore, different DMI may affect rumen microbial function, with higher DMIs usually promoting microbial amino acid metabolism^35^. In our study, FF had slightly lower DMI than CF. Thus, the enhanced AA and carbohydrate metabolism in the FF rumen microbiota was not caused by DMI, but by changes in the active microbial community and in the nutritional value of the fermented feed.

The *Leuconostoc lactis* and *Lactococcus lactis* are the dominant species contributing to carbohydrate metabolism. *Leuconostoc lactis* has been engineered to synthesize a variety of oligosaccharides and has shown potential in carbohydrate metabolism^36^. The *Lactococcus lactis* is most widely known for its association with the milk environment and in the production of dairy products. Furthermore, *Lactococcus spp*. also have potential for animal feeds compared with commercial synbiotic consortia^28,37^. In our study, the increased AA concentration in the feed may be attributed to the proteolytic system of *Lactococcus lactis*, which is associated with its ability to degrade casein in milk^38^. Overall, our study demonstrated that the target microorganisms added for feed fermentation are not necessarily present in the final fermented feed. However, they can improve the nutritional quality of the feed by increasing the concentration of free AA and promoting the pre-decomposition of complex carbohydrates.

After clarifying the nutritional value and microbiota of the fermented feed, we fed it to kids to assess its in vitro application effect. Firstly, our results revealed that feeding fermented feed could increase the body and carcass weights of the kid during the lactation stage, with no impact on organ weights or the lengths or volumes of each digestive tract segment. It means that fermented feed promoted skeletal muscle development, consistent with previous feeding experiments^39^. In the present study, kids were not housed or fed individually, resulting in a lack of individual feed intake data. Therefore, it cannot be ruled out that fermented feed might increase the feed intake by accelerating the passage rate of the diet in the digestive tract^40^. A fistulated goat can be used to investigate the in vivo digestion of fermented diets in the future. Research on sheep nutrition has advanced from basic investigations of protein requirements to the in-depth study of AA requirements^41^. Additionally, several previous studies have shown that increasing AA in the feed can enhance slaughter weight, which is associated with AA balance and the fact that AA serve as the raw material for muscle synthesis, such as lysine and methionine^42,43^. Furthermore, Ca and P concentrations increased in fermented feed, which also supports carcass development^44^. Studies on fermented feed used in calves and lambs are scarce, whereas research on piglets has shown that it improves growth performance and nutrient digestibility^26^. Jia et al.^11^ indicated that the combination of *Bacillus licheniformis* and *Saccharomyces cerevisiae* feeding improved average daily gain and feed conversion efficiency in fattening lambs, with effects superior to those of the single-strain supplementation groups. Feeding beneficial microbes to promote feed digestion in the rumen may differ from the use of in vitro pre‑fermented feed. Hence, our study demonstrates that the nutritional characteristics of fermented feed were significant for kids, as they promote kid growth by providing additional AA, Ca, and P.

Except for the nutritional value, viable microorganisms in the fermented feed also changed significantly in our study. It may interact with gastrointestinal microbiota. Also, there is an interaction between dietary AA and the gastrointestinal microbiota of young ruminants^45,46^. However, the microbial interaction network found that functional cooperative relationships among species have become closer, which was consistent with changes in microbiota function. This is partly due to the metabolic redundancy of core functional rumen microbiota^47^, which can adapt to fermented feed without the addition or elimination of species. Furthermore, fermented feed provides more nutrients to enhance the frequency of metabolic interactions among various microorganisms. Increased interactions have been observed across multiple experiments using high-nutrient substrates for the rumen microbiota^48,49^. The Firmicutes C was the genus with the second-highest contribution in the FF group, instead of Firmicutes A in the CF group. Furthermore, these taxa played significant roles in their networks and had more associations with other taxa. It means that fermented feed promoted the expansion of special taxa and supported the feed digestion. Although the degree of dietary fermentation by different microorganisms varies, previous studies have shown that carbohydrate and AA metabolism pathways are upregulated^50,51^. This is consistent with the results of the present study, which collectively indicate that fermented diets promote the metabolic function of the rumen microbiota.

The gene expression in rumen epithelium is closely related to dietary composition. In our study, down-regulated pathways included protein digestion and absorption, and fat digestion and absorption. However, the rumen epithelium plays a relatively minor role in the breakdown of the diet but a significant role in nutrient transport^52^. The energy metabolism was enhanced in the kid fed fermented feed. Furthermore, a recent study showed that tryptophan could promote early rumen development^53^. In the present study, increased tryptophan metabolism also indicates accelerated rumen epithelial development, which is consistent with the results of enhanced mitochondrial energy metabolism. Hence, our findings suggest that fermented feed not only equips the rumen epithelium with adaptive capacity to dietary changes but also enhances its energy supply, thereby underpinning essential epithelial functions, including nutrient absorption, cell proliferation, and repair.

This study has certain limitations to note. First, selecting only one Qingshan goat kid per pen for slaughter may introduce selection bias. Although we strictly selected homogeneous Qingshan goat kids from a large flock for the trial at the outset, and this approach is commonly adopted in sheep experiments^11,46^, it may still carry potential selection bias. Future studies could conduct feeding trials with various fermented feeds and use a larger number of experimental kids to replicate and validate the results and conclusions of the present study.

In conclusion, a novel fermentation consortium (*Saccharomyces cerevisiae*, *Bacillus licheniformis*, and *Enterococcus faecalis*) was developed, and its fermentation conditions were optimized. Analysis of the fermented diet showed the consortium exerted a synergistic effect on boosting nutritional value (increased crude protein and free AA concentrations), with low abundance of initially added strains and emergence of a distinct new microbial community. Goat kid feeding trials confirmed that this fermented diet significantly improved growth and carcass performance, which was mechanistically linked to enhanced rumen epithelial energy metabolism and the promotion of dietary nutrient decomposition by rumen microbiota. This study proposes a three-strain combined fermentation mode for increasing the nutrient conversion efficiency of feed and partially reveals the mechanism by which fermented diets promote kid growth via enhancing rumen microbial amino acid synthesis and rumen epithelial mitochondrial energy metabolism. Future studies should investigate the combined effects of different strains in fermented diets to enhance fermentation efficiency continuously.

## Methods

### Preparation of microorganisms

The rumen content was obtained from one healthy adult fistulated Qingshan goat (18 months old, 35.2 kg body weight). Upon completion of cultivation, 2 mL of each bacterial suspension was aspirated using a sterile pipette tip and transferred into a sterile preservation tube for strain identification (Shanghai Personal Biotechnology Co., Ltd, Shanghai, China). After purification, single colonies were inoculated into liquid medium for cultivation (Beijing Solarbio Science & Technology Co., Ltd, Beijing, China). The total amount of DNA was detected by fluorescent dye (Quant-iT PicoGreen dsDNA Assay Kit, Thermo Fisher Scientific (China) Inc., Shanghai, China). The standard Illumina TruSeq Nano DNA LT library preparation experimental process (Illumina TruSeq DNA Sample Preparation Guide, Illumina, Inc., California, USA) was used to construct the genomic library. The standard PacBio Template Prep Kit 1.0 library preparation process (20 kb Template Preparation Using BluePippin Size Selection, Pacific Biosciences of California, Inc., California, USA) was used to construct the genomic host library. The sequencing process was performed according to previously described methods^54^. The genome information of these microorganisms was presented in Supplementary Table 4.

### Preparation of microbially fermented feed

The feed ingredients composing the starter feed included corn, soy flour, soybean hulls, alfalfa meal, and premix (Supplementary Table 5). First, the optimal microorganism addition ratio for fermented feed was evaluated through an in vitro fermentation experiment by comparing nutritional value. Feedstuffs of equal weight were mixed thoroughly, and different proportions of each microorganism were added to the feed and homogenized. Subsequently, the feed was fermented using one-way fermentation bags (Shanghai Personal Biotechnology Co., Ltd, Shanghai, China). The experimental design was generated using the Orthogonal Design Assistant software (V 3.1), consisting of 10 groups (Supplementary Table 6). We used the L9(3³) orthogonal array, a saturated design for evaluating main effects. No significant interaction was assumed in the present study. The 1~3% supplementation concentration gradient of the microbial consortium for fermenting corn, corn silage, and soybean meal was set according to Guo et al.^55^ and significant differences were observed within this gradient, with the optimal concentration of each strain falling in 1~3%. A total of 6 replicates were arranged for each treatment. After fermentation, the detection method of crude protein (CP), neutral detergent fiber (NDF), and acid-soluble protein (AP) was referenced from a previous study^11^. The enzymatic hydrolysis efficiency (E) was calculated using the following formula^56^:

$$E=0.53\times {CP}+0.13\times {NDF}+0.34\times {AP}$$

For each strain, the mean E value of all groups containing the same addition level was calculated separately. The addition level with the highest mean E was selected as the optimal level for that strain. The theoretical optimal combination of strains was obtained by combining the optimal level of each strain, which may not correspond to any individual group included in the orthogonal array. The ratio with the highest range was considered the most limiting factor. The feeds in the CON group refer to those without microbial inoculation.

After determining the optimal addition ratio, we investigated the optimal fermentation conditions, including fermentation temperature, moisture content, aerobic fermentation time, and anaerobic fermentation time, consisting of 10 groups (Supplementary Table 7). After fermentation, the optimal fermentation condition was filtered by comparing the E as above. The nutritional value indices included CP, crude fat, crude fiber, crude Ash (Ash), calcium (Ca), phosphorus (P), and amino acid (AA) composition as previously described in a study^57^. The feeds in the CON group refer to those without microbial inoculation and under non-fermentation conditions.

### Viable microbiome composition of fermented feed

The strain inoculation ratio and fermentation conditions for the preparation of fermented feed in the formal experiment were determined based on the optimal parameters screened from preliminary orthogonal experiments. To compare the advantages of fermented feed over conventional feed, we determined the nutrient contents of three feed types: conventional feed (CF), fermented feed (FF), and microorganism-added unfermented feed (MAUF). The detection indicators included CP, NDF, AP, and AA composition. The dry matter content of FF and CF was 72.29% and 89.12%. The method for AA determination was referenced from previous studies^58^. After fermentation, we stored the feed immediately at -80 °C to detect the viable microbial composition and function via metatranscriptomic sequencing. Total RNA was extracted using the RNA PowerSoil® Total RNA Isolation Kit (12866-25) (MoBio, USA) according to the manufacturer’s instructions. Each library was sequenced by the Illumina NovaSeq platform (Illumina, USA) with a PE150 strategy at Personal Biotechnology Co., Ltd. (Shanghai, China). The sequencing process was performed according to previously described methods^59^.

The feed was also used for absolute quantification (AQ) of the bacteria using AQ-PCR. DNA in the rumen samples was extracted using the Mag-Bind Soil DNA Kit (200) (Cat: M5635-02, Omega Bio-tek, Inc., GA, USA). The concentration and purity of microbial DNA were measured using ND-2000 (Thermo Fisher Scientific Inc., MA, USA). AQ-PCR was performed as described by Adebayo et al.^60^ The forward and reverse primers were as follows: 5′-ACTCCTACGGGAGGCAG-3′ (F) and 5′- GACTACCAGGGTATCTAATC-3′ (R). The final copy numbers of individual microbes per gram of dry matter of solid contents were expressed.

### Goat kids feeding experiment

The goat kid feeding experiment was conducted on a commercial farm in Heze, China. A total of 48 healthy male Qingshan goat kids were divided into 12 pens, with four kids per pen, and each group included six pens. The experimental units were the pens (*n* = 6). Kids in the CF group (initial body weight: 3.11 ± 0.07 kg; initial age: 20 days) were fed CF, while kids in the FF group (initial body weight: 3.17 ± 0.10 kg; initial age: 20 days) were fed fermented feed. The experiment lasted 40 days, and the kids had *ad libitum* access to starter feed and water throughout the day (Dry matter intake, DMI: CF, 82.5 ± 11.04 g/d; FF, 81.7 ± 13.35 g/d). All experimental procedures and protocols were approved by the Research Committee of Jiangsu Academy of Agricultural Sciences following the regulations for the Administration of Affairs Concerning Experimental Animals (Decree No. 101 of Jiangsu Academy of Agricultural Science). One kid in a single pen of the FF group sustained a minor, non-infectious accidental injury confined to this individual only. As the pen was the experimental unit, the entire pen was excluded from statistical analysis to ensure data reliability.

The body weight (BW) of all kids was measured on the initial day (Day 1) and the last day (Day 40) of the feeding trial. One kid with body weight close to the average was selected from each pen for slaughter at the end of the experiment, and used to determine the slaughter performance. Live BW was recorded, and goats were anesthetized with sodium pentobarbitone, euthanized via jugular vein exsanguination, and then skinned and eviscerated in accordance with the previous study^61^. Internal organs (heart, liver, spleen, lungs, and kidneys) were weighed. Hot carcass weight (CW) was determined. Empty weights of the rumen, reticulum, omasum, and abomasum were recorded, while rumen volume was measured by filling the rumen with water. The lengths of the small intestinal segments were recorded. Fecal samples from each kid were collected by the point collection method to estimate the apparent digestibility of feed to reveal the resource utilization efficiency^62^. Feed and fecal samples were dried in the oven at 65 °C for 48 h, and the dried feed and fecal samples were crushed and stored until use for determining acid-insoluble ash (AIA). The digestibility of feed was calculated according to the formula^62^:

$${Digestibility}\left( \% \right)=[1-({Ad}\times {Nf}/{Af}\times {Nd})]\times 100$$

Where Ad is the AIA in feed, Af is the AIA in feces, Nd is the nutrients in feed, and Nf is the nutrients in feces. Organic matter (OM), CP, ether extract (EE), NDF, and acid detergent fiber (ADF) contents of feed and feces were determined.

After the rumen was collected and washed with phosphate-buffered saline, three pieces of rumen tissue from the ventral sac were collected and stored for analysis of transcriptomics. Total RNA was isolated using TRIzol reagent (Invitrogen Life Technologies, Shanghai, China). The sequencing library was then sequenced on the NovaSeq 6000 platform (Illumina), Shanghai Personal Biotechnology Co., Ltd, according to the previous study^58^. The volatile fatty acid (VFA) concentrations in rumen content were analyzed using an Agilent G689N gas chromatograph and an Agilent G7683 autosampler (Agilent Technologies, Inc., CA, USA) as described in a previous study^58^.

For rumen content microbiota, DNA was extracted using a DNeasy PowerSoil Kit (Cat. No.12888, Qiagen, Valencia, CA, USA). The extracted total DNA was processed to construct metagenome shotgun sequencing libraries using Illumina TruSeq Nano DNA LT Library Preparation Kit (Illumina, USA) according to the previous study^58^.

### Statistical analysis

For the nutritional value of feed, the data were analyzed using one-way analysis of variance (ANOVA) with Tukey’s test. The feed utilization efficiency and growth performance data were analyzed using a t-test. A *P*-value < 0.05 was considered statistically significant. The PCA analysis was used to show the distribution of AA composition. Data on AA concentrations were normalized using the Z-score method, and heatmap visualization was performed via the Genescloud platform (https://www.genescloud.cn).

For metatranscriptomic data on the feed, transcripts per million (TPM) for each species and gene-level α diversity for each sample were calculated and then compared using the Wilcoxon test, with *P* < 0.05 considered significant. We used Procrustes analysis to show the significance of group separation using Principal Coordinates Analysis (PCoA). The composition and function of viable microorganisms were normalized using the Z-score method and compared using the Wilcoxon test. The heatmap was generated using the Genescloud platform (https://www.genescloud.cn). The significantly different metabolism pathways on KEGG L3 were included in network analysis based on metabolic relationships. The neutral community model (NCM) was used to quantify the contribution of stochastic processes. The R² represents the goodness-of-fit of the NCM, reflecting the degree to which the model explains microbial community assembly. The NCM was considered to have failed when the model fit R² was less than 0.1 (or negative, indicating a worse fit than a null model).

For metagenomics data on rumen content, the network topology index and gene-level α diversity for each sample were calculated and then compared using the Wilcoxon test, with *P* < 0.05 considered significant. For microbial co-occurrence network analysis, the following parameters were set: taxonomic level: species; Removal threshold for taxonomic features: detected in ≤3 samples; Abundance threshold for taxonomic features: > 0.01%; Correlation algorithm: SparCC; Edge filtering threshold: R threshold = 0.6. Species-level differences were identified using linear discriminant analysis effect size (LEfSe), with thresholds set at a Linear Discriminant Analysis (LDA) score >2.0 and *P* < 0.05 to detect significant differences in species between groups.

For the transcriptome, differential expression of genes was analyzed by DESeq2 (v1.38.3) with screened conditions as follows: expression difference multiple |log2FoldChange|> 1, significant *P*-value < 0.05. The gene co-occurrence network was generated under the condition that the protein-protein interaction network score was greater than 0.95. Then, the metabolism pathways that were significantly down-regulated and up-regulated on KEGG L3 were included in network analysis based on metabolic relationships.

## Supplementary information

Supplementary information

## Data Availability

The datasets analysed during the current study are available in the National Information Center of China (GSA, Project No.: PRJCA048672).
