# Supplementary material for: Fermented feed-rumen microbiota axis mediates goat kid growth via microbial amino acid synthesis and ruminal epithelial energy metabolism
Source: NPJ Biofilms Microbiomes. 2026 Jun 11;12:184. doi: 10.1038/s41522-026-01046-z (PMC13612522; doi:10.1038/s41522-026-01046-z)
Supplement: Supplementary file 1 [file 41522_2026_1046_MOESM1_ESM.pdf]

Fermented feed-rumen microbiota axis mediates goat kid growth via microbial amino acid synthesis and ruminal epithelial energy metabolism

Fanlin Kong [a,b,c,†], Fenghui Liu [a,b,c,†], Yong Qian [a,b,c], Ziyu Wang [d], Wenfeng Zhang [a], Yan Wang [a], Jianli Zhang [a,b,c], Yinxia Li [a,b,c], Jun Zhang [a,b,c], Shuo Wang [e], Dongwen Dai [f], Zhiping Lin [g], Huili Wang [a,b,c], Chunhua Meng [a,b,c,*]

[a] Institute of Animal Science, Jiangsu Academy of Agricultural Sciences, Nanjing 210014, China
[b] Key Laboratory of Crop and Animal Integrated Farming, Ministry of Agriculture and Rural Affairs, 210014 Nanjing, China
[c] Jiangsu Provincial Engineering Research Center of Precision Animal Breeding, 210014 Nanjing, China
[d] College of Animal Science, Nanjing Agricultural University, 210014 Nanjing, China
[e] Inner Mongolia Agricultural University, Key Laboratory of Dairy Biotechnology and Engineering, Ministry of Education, Hohhot 010018, China
[f] Academy of Animal Science and Veterinary, Qinghai University, Xining 810016, China
[g] Nanjing Youyuan Dairy Research Institute Co., Ltd., Nanjing 211100, China

[*] Address correspondence to: Chunhua Meng (mengchunhua@jaas.ac.cn)
[†] These authors contributed equally to this work

E-mail: Fanlin Kong (fanlinkong1126@163.com);
Fenghui Liu (18464038729@163.com);
Yong Qian (19970019@jaas.ac.cn);
Ziyu Wang (wangizyu@njau.edu.cn);
Wenfeng Zhang (2210848482@qq.com);
Yan Wang (18205508791@163.com);
Jianli Zhang (20030017@jaas.ac.cn);
Yinxia Li (20130016@jaas.ac.cn);
Jun Zhang (20040011@jaas.ac.cn);
Shuo Wang (wsnmgcow@163.com);
Dongwen Dai (2025990090@qhu.edu.cn);
Zhiping Lin (linzp@wgdairy.com.cn);
Huili Wang (wanghuili318@163.com)

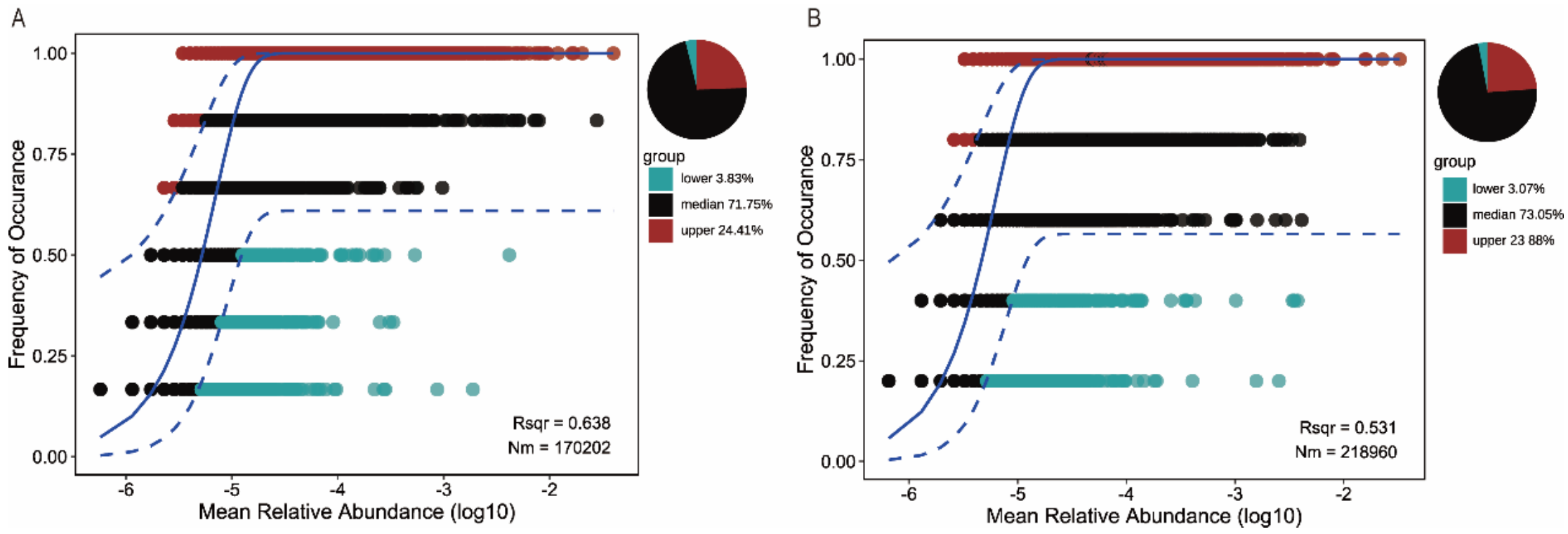

Supplementary Figure 1. Neutral community model of rumen microbiota of kids fed the conventional feed (A) and fermented feed (B).

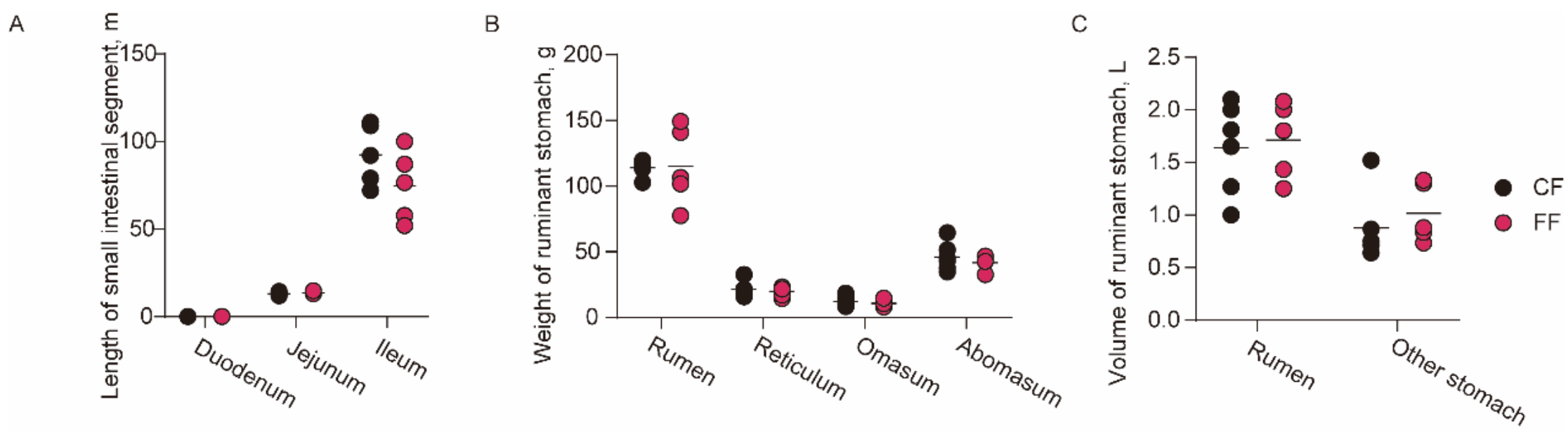

Supplementary Figure 2. Length, weight, and volume of segments of the gastrointestinal tract.

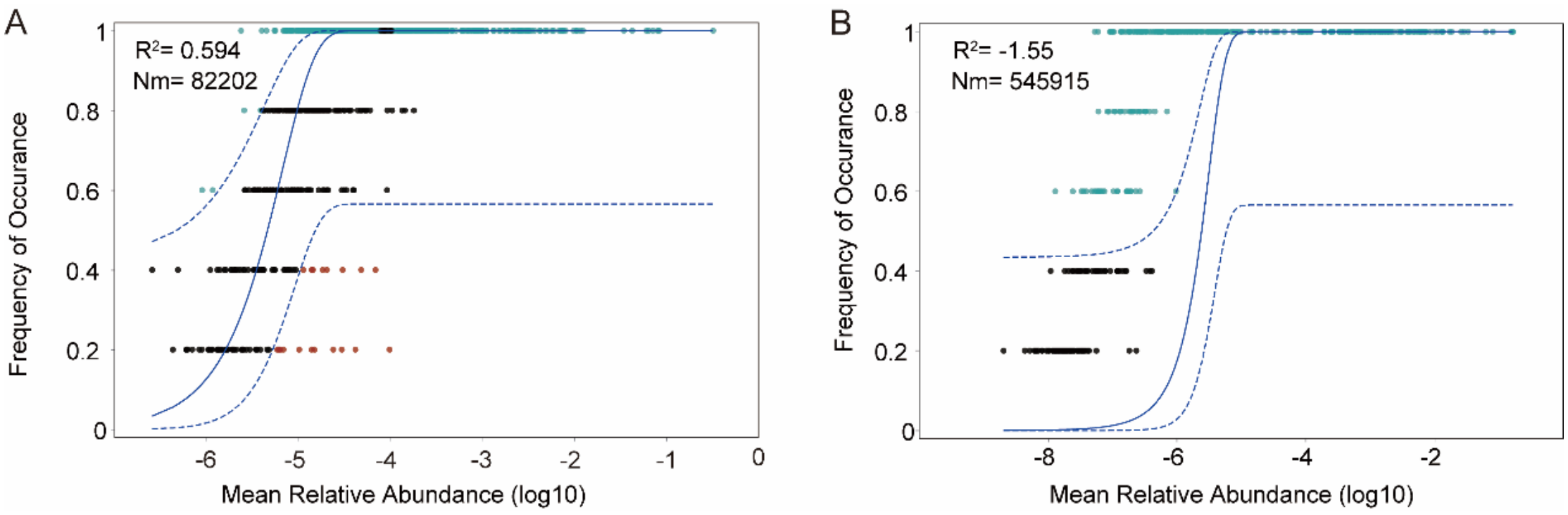

Supplementary Figure 3. Neutral community model of viable microbiota in the conventional feed (A) and fermented feed (B).

Supplementary Table 1. The calculation process of the optimal microorganism addition amount

Items	*Bacillus licheniformis*	*Enterococcus faecalis*	*Saccharomyces cerevisiae*	E
A	1	1	1	22.47
B	1	2	2	22.21
C	1	3	3	22.78
D	2	1	2	26.44
E	2	2	3	27.2
F	2	3	1	26.88
G	3	1	3	24.09
H	3	2	1	22.48
I	3	3	2	22.04
1% Average	22.49	24.33	23.94	
2% Average	26.84	23.96	23.56	
3% Average	22.87	23.90	24.69	
Range	4.35	0.43	1.13	
Optimal level, %	2	1	3	

Supplementary Table 2. The calculation process of the optimal fermentation conditions

Items	Aerobic time, h	Anaerobic time, h	Temperature, ℃	Moisture content, %	E
J	24	3	30	35	16.80
K	24	5	33.5	40	18.49
L	24	7	37	45	18.08
M	36	7	33.5	35	18.57
N	36	3	37	40	19.29
O	36	5	30	45	19.85
P	48	5	37	35	18.33
Q	48	7	30	40	19.50
R	48	3	33.5	45	18.67
Low	17.79	17.9	18.26	18.72	
Medium	19.24	19.1	18.9	18.58	
High	18.84	18.87	18.72	18.57	
Range	1.45	1.94	0.64	0.15	
Optimal level	36	5	33.5	35	

Supplementary Table 3. Detailed information on significantly different species (SDS) between FF and CF

SDS	Domain	Genus	*P*-value	Log_2(FC)
Citrobacter europaeus	Bacteria	Citrobacter	0.02	-13.33
Latilactobacillus curvatus A	Bacteria	Latilactobacillus	0.02	-12.95
Leuconostoc falkenbergense	Bacteria	Leuconostoc	0.02	-12.65
Lactococcus A chungangensis	Bacteria	Lactococcus A	0.02	-12.14
Leuconostoc citreum	Bacteria	Leuconostoc	0.02	-10.55
Enterococcus G italicus	Bacteria	Enterococcus G	0.02	-9.72
Aspergillus glaucus	Eukaryota	Aspergillus	0.02	-7.42
Fructilactobacillus fructivorans	Bacteria	Fructilactobacillus	0.02	-3.93
Brevibacillus B laterosporus	Bacteria	Brevibacillus B	0.02	-3.08
Lactobacillus delbrueckii	Bacteria	Lactobacillus	0.02	-2.41
Escherichia sp005843885	Bacteria	Escherichia	0.02	-1.80
Pantoea agglomerans	Bacteria	Pantoea	0.02	-1.73
Acinetobacter brisouii A	Bacteria	Acinetobacter	0.02	-1.34
Saccharomyces cerevisiae	Eukaryota	Saccharomyces	0.02	-1.03
Aspergillus cristatus	Eukaryota	Aspergillus	0.02	-0.98
Escherichia fergusonii	Bacteria	Escherichia	0.02	-0.82
Lactobacillus delbrueckii B	Bacteria	Lactobacillus	0.02	-0.08
Lactococcus cremoris	Bacteria	Lactococcus	0.02	-0.04
Limosilactobacillus fermentum	Bacteria	Limosilactobacillus	0.02	0.11
Leuconostoc holzapfelii	Bacteria	Leuconostoc	0.02	0.22
Acinetobacter johnsonii	Bacteria	Acinetobacter	0.02	0.37
Lactococcus A raffinolactis	Bacteria	Lactococcus A	0.02	0.61
Escherichia coli	Bacteria	Escherichia	0.02	1.06
Lactococcus hircilactis	Bacteria	Lactococcus	0.02	1.06
Weissella confusa B	Bacteria	Weissella	0.02	1.34
Leuconostoc lactis A	Bacteria	Leuconostoc	0.02	1.34
Lactococcus lactis	Bacteria	Lactococcus	0.02	1.39
Achromobacter denitrificans	Bacteria	Achromobacter	0.02	1.41
Aspergillus ruber	Eukaryota	Aspergillus	0.02	1.41
Wallemia mellicola	Eukaryota	Wallemia	0.02	1.65
Aspergillus chevalieri	Eukaryota	Aspergillus	0.02	1.65
Escherichia sp004211955	Bacteria	Escherichia	0.02	1.71
Escherichia sp002965065	Bacteria	Escherichia	0.02	1.76
Escherichia marmotae	Bacteria	Escherichia	0.02	2.76
Weissella confusa	Bacteria	Weissella	0.02	3.26
Trichoderma harzianum	Eukaryota	Trichoderma	0.02	3.48
Acinetobacter lanii	Bacteria	Acinetobacter	0.02	4.15
Phaffia rhodozyma	Eukaryota	Phaffia	0.02	4.41
Leuconostoc lactis	Bacteria	Leuconostoc	0.02	4.85
Lacticaseibacillus manihotivorans	Bacteria	Lacticaseibacillus	0.02	4.97
UMGS1491 sp017432165	Bacteria	UMGS1491	0.02	5.36

Latilactobacillus sakei	Bacteria	Latilactobacillus	0.02	5.37
Monilinia narnavirus H	Viruses	Unclassified_f_Narnaviridae	0.02	8.95
Lactococcus sp009601055	Bacteria	Lactococcus	0.02	11.78
Faecalibacteriu m sp014858325	Bacteria	Faecalibacterium	0.02	29.90

Supplementary Table 4. Overview of Genome Assembly Results

Microorganism	Reference	ANI, %	Genome size, Mb	Completeness, %	ORF num
Bacillus licheniformis	GCF_000011645.1	99.64	4.47	98.82	4,858
Enterococcus faecalis	GCF_029024925.1	98.81	3.21	99.63	2,735
Saccharomyces cerevisiae	KF447149	99.83	13.02	99.00%	6,005

Supplementary Table 5. The ingredient and nutritional compositions of kid starter feed.

Items	Composition
Ingredients, % dry matter basis	
Corn	25
Soy flour	29
Protein balls	23.5
Alfalfa meal	10
Soybean hulls	10
Premix	2.5
Nutrition, dry matter basis	
Moisture content, %	89.12
Crude protein, %	23.21
Neutral detergent fiber, %	27.48
Acid-soluble protein, %	0.70
Total amino acids, μg/g	330
Lysine, μg/g	0.33
Methionine, μg/g	1.67

Supplementary Table 6. The addition proportions of each microorganism in different groups

Items	A	B	C	D	E	F	G	H	I	CON
Bacillus licheniformis, %	1	1	1	2	2	2	3	3	3	0
Enterococcus faecalis, %	1	2	3	1	2	3	1	2	3	0
Saccharomyces cerevisiae, %	1	2	3	2	3	1	3	1	2	0

Supplementary Table 7. The experiment design of optimal fermentation conditions

Items	J	K	L	M	N	O	P	Q	CON
fermentation temperature, ℃	30	33.5	37	33.5	37	30	37	30	33.5
moisture content, %	35	40	45	35	40	45	35	40	45
aerobic fermentation time, h	24	24	24	36	36	36	48	48	48
anaerobic fermentation time, h	3	5	7	7	3	5	5	7	3
